# The Role of the Spleen and the Place of Splenectomy in Autoimmune Hemolytic Anemia—A Review of Current Knowledge

**DOI:** 10.3390/diagnostics13182891

**Published:** 2023-09-09

**Authors:** Zorica Cvetković, Nikola Pantić, Mirjana Cvetković, Marijana Virijević, Nikica Sabljić, Gligorije Marinković, Vladimir Milosavljević, Zlatko Pravdić, Nada Suvajdžić-Vuković, Mirjana Mitrović

**Affiliations:** 1 Department of Hematology, University Hospital Medical Center Zemun, 11080 Belgrade, Serbia; 2 Medical Faculty, University of Belgrade, 11000 Belgrade, Serbia; 3 Clinic for Hematology, University Clinical Center of Serbia, 11000 Belgrade, Serbia; 4 Department for HPB Surgery, University Hospital Medical Center Bežanijska Kosa, 11070 Belgrade, Serbia

**Keywords:** autoimmune hemolytic anemia, spleen, diagnostics, splenectomy, treatment

## Abstract

Autoimmune hemolytic anemia (AIHA) is a rare, very heterogeneous, and sometimes life-threatening acquired hematologic disease characterized by increased red blood cell (RBC) destruction by autoantibodies (autoAbs), either with or without complement involvement. Recent studies have shown that the involvement of T- and B-cell dysregulation and an imbalance of T-helper 2 (Th2) and Th17 phenotypes play major roles in the pathogenesis of AIHA. AIHA can be primary (idiopathic) but is more often secondary, triggered by infections or drug use or as a part of other diseases. As the location of origin of autoAbs and the location of autoAb-mediated RBC clearance, as well as the location of extramedullary hematopoiesis, the spleen is crucially involved in all the steps of AIHA pathobiology. Splenectomy, which was the established second-line therapeutic option in corticosteroid-resistant AIHA patients for decades, has become less common due to increasing knowledge of immunopathogenesis and the introduction of targeted therapy. This article provides a comprehensive overview of current knowledge regarding the place of the spleen in the immunological background of AIHA and the rapidly growing spectrum of novel therapeutic approaches. Furthermore, this review emphasizes the still-existing expediency of laparoscopic splenectomy with appropriate perioperative thromboprophylaxis and the prevention of infection as a safe and reliable therapeutic option in the context of the limited availability of rituximab and other novel therapies.

## 1. Introduction

Autoimmune hemolytic anemia (AIHA) is an acquired autoimmune disease characterized by the increased destruction of autologous red blood cells (RBCs) due to the presence of immunoglobulin (Ig)G, IgM, IgA, or complements (usually C3d) bound to RBC membrane antigens [1,2,3]. Although this rare, very heterogeneous, and sometimes life-threatening condition occurs across all age groups, with a reported annual incidence of 1–3 per 100,000 persons, the incidence of chronic and relapsing cases increases with age (i.e., over the age of 40) [2].

Shortened RBC survival due to hemolysis clinically manifests in symptoms which include weakness, dyspnea, jaundice, acrocyanosis or Raynaud phenomena, and splenomegaly, and laboratory tests reveal normocytic/macrocytic anemia, reticulocytosis, and elevated indirect (unconjugated) bilirubin and lactate dehydrogenase with reduced or fully consumed haptoglobin. The spherocytosis, agglutination, and polychromasia of RBCs can be seen in a peripheral blood smear. Hemoglobinuria and the presence of urinary hemosiderin indicate the onset of extravascular hemolysis [3].

The severity of AIHA is determined by the type and affinity of autoantibodies (autoAbs), which reduce RBC lifespan, and by the capacity of bone marrow to compensate for hemolysis. A diagnosis of hemolytic anemia is issued when RBC survival drops below 100 days [4]. The spleen plays a major role in the pathobiology of AIHA, both in the synthesis of autoAbs and in the immune destruction of RBCs.

## 2. Diagnostic Workup, Serological Features, and the Classification of AIHA

The diagnostic hallmark of AIHA is a positive direct Coombs test (antiglobulin test—DAT), and the classification of AIHA into warm AIHA (wAIHA) and cold AIHA (cAIHA) is based on the serological and thermal characteristics of the detected autoAbs. The most common type of AIHA is wAIHA, which accounts for 60–70% cases and is mediated by the autoreactive polyclonal IgG that binds to RBC antigens maximally at 37 °C. The most frequently occurring type of cAIHA, and the second most frequent type of AIHA (20% of all AIHA cases), is cold agglutinin disease (CAD). CAD is mediated in 90% of patients by oligoclonal or clonal IgM autoAbs that maximally react at 4 °C and strongly activate complement due to their high density on RBC surfaces and the structure of IgM [5]. A rare subtype of cAIHA is paroxismal cold hemoglobinuria (PCH). This represents only 1% of all AIHA cases, is usually seen in childhood, and is characterized by transient hemolysis triggered by an upper respiratory tract infection. PCH is defined by DAT positivity only for the complement and the presence of biphasic Donath–Landsteiner Abs, which adsorb and fix the early components of the complement system on the RBC membrane at low temperatures and afterwards, with warming, dissociate from the RBC, leading to the activation of downstream complement cascade effects, the formation of a lytic complex (MAC–membrane attack complex), and the onset of intravascular hemolysis [6].

In 5–10% of patients with the laboratory characteristics of both wAIHA and CAD, the diagnosis of mixed-type AIHA is established [7]. Furthermore, wAIHA can concomitantly or sequentially be associated with other autoimmune cytopenias (thrombocytopenia and, rarely neutropenia), the conditions usually assigned as Evans syndrome [8].

**Figure 1:**
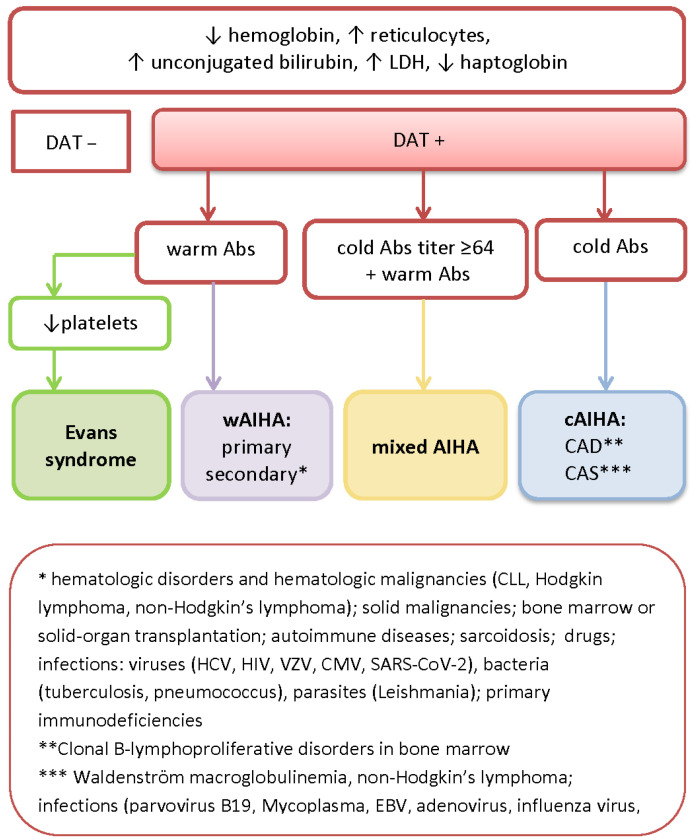
Simplified algorithm for the diagnosis of AIHA. Abbreviations: LDH—lactate dehydrogenase; DAT—direct antiglobulin test; Abs—antibodies; TMA—thrombotic microangiopathy; TTP—thrombotic thrombocytopenic purpura; HUS—hemolytic uremic syndrome; DIC—disseminated intravascular coagulation; wAIHA—warm autoimmune hemolytic anemia; cAIHA—cold autoimmune hemolytic anemia; CAD—cold agglutinin disease; CAS—cold agglutinin syndrome; CLL—chronic lymphocytic leukemia; HCV—hepatitis C; HIV—human immunodeficiency virus; CMV—cytomegalovirus; EBV—Epstein–Barr virus.

Simplified laboratory algorithms for AIHA diagnosis and the classification of AIHA are shown in Figure 1 and Table 1 according to the autoAb istotype and the temperature at which the autoAbs show maximal binding.

It is important to notice that DATs may be negative in nearly 10% of patients with AIHA, predominantly those with wAIHA. This usually happens if the affinity and density of the autoAbs on the RBC surface is low (i.e., less than 500 autoAbs), or because of the presence of IgA [9]. On the other hand, false-positive Coombs test results are seen in the presence of antiphosholipid Abs (lupus anticoagulant and anti-cardiolipin Abs) due to cytophilic or non-specifically adsorbed IgG being on the surface of RBCs, and the eluates of RBCs can determine whether the Abs were immunologically or nonspecifically attached to these cells [10,11]. False-positive Coombs test results are also seen if the IgG in the serum is elevated after the transfusion of RBCs; after the administration of intravenous Ig (IVIG), monoclonal antiCD38Ab (daratumumab), and some other therapies; and even in healthy blood donors [5,12,13]. In patients successfully treated for AIHA, DAT may remain persistently positive [5]. DAT false positives and DAT false negatives in atypical AIHA highlight the necessity and the importance of careful clinical and serological correlation, especially given that evidence of hemolysis is necessary for AIHA diagnosis.

AIHA is idiopathic (primary) in less than 50% of cases. In the remaining patients, the underlying diseases are identified, the most common being hematological malignancies, solid tumors, autoimmune and inflammatory diseases, and immunodeficiencies. A high incidence of AIHA is also observed in transplant recipients. AIHA can be triggered by acute and chronic bacterial and viral infections, including SARS-CoV-2, and numerous drugs [5,14]. Of particular interest is the distinction between primary and secondary CAD. Primary CAD is defined as the absence of underlying diseases, excluding clonal B-lymphoproliferative disorders of the bone marrow in the absence of clinical and radiological evidence of active disease, while the presence of cold agglutinins in the course of apparent malignancy or infection is defined as cold agglutinin syndrome (CAS) [3]. Severe AIHA implies a drop in hemoglobin (Hb) to a level below 80 g/L and the need for transfusions at intervals of ≤7 days [5].

## 3. The Role of the Spleen and Bone Marrow in the Immunological Background of AIHA

### 3.1. Spleen as the Place of Origin of Autoantibodies in AIHA

Hitherto, the pathogenesis of AIHA has not been fully understood. Today, it is believed that, as in autoimmune and lymphoproliferative diseases, the breakdown of immunological central and peripheral tolerance, T- and B-cell dysregulation, and a shift towards T-helper 2 (Th2) and Th17 phenotype play major roles in the generation of autoAbs directed against RBCs.

The spleen is the largest secondary lymphoid organ and plays an important role in host defense but also in autoimmunity. The spleen is divided by function and structure into red and white pulp, with a perifollicular (marginal) zone in between. The primary immunological region of the spleen comprises white pulp, which occupies less than a quarter of splenic tissue. Unlike the lymph nodes, the spleen lacks afferent lymphatic vessels, and therefore, all cells and antigens enter the spleen via the blood [15]. The secretion of Abs by lymphoid follicle germinal center (GC) plasma cells (PCs) that have lifespans of 2–3 days is tightly regulated by negative feedback interactions with follicular Th cells (Tfh). Tfh cells produce IL-21, a protein critical for the processes of affinity maturation, GC longevity and function, and B-lymphocyte terminal differentiation [16]. Conventional PCs are designated to participate in bone marrow homing. Embedded in bone marrow niches, PCs may survive as long-lived PCs (LLPCs), persisting for up to the lifetime of the hosts in the absence of repeated antigen stimulation, together with memory CD4+ and memory CD8+ T cells [17,18]. It has been shown that, unlike conventional PCs, autoimmune LLPCs that secrete high-affinity IgG autoAbs abnormally accumulate in the spleen and show positive feedback with Tfh cells [19,20,21,22]. In the recently published work of Zeng et al., splenic white pulp fibroblastic reticular cells (FRCs) are recognized as a key factor that control autoreactive B-cell responses through CD36-mediated lipid uptake and the consequently enhanced mitochondrial oxidative phosphorylation in B cells [22].

The association between autoimmune disorders and neoplastic diseases, especially lymphoid neoplasms of B-cell origin (chronic lymphocytic leukemia(CLL), B-cell non-Hodgkin’s lymphoma, and Hodgkin’s lymphoma), was noted a long time ago. Autoimmune diseases, predominantly rheumatological (systemic lupus erythematosus, rheumatoid arthritis, and ankylosing spondylitis), endocrinological (type-1 insulin-dependent diabetes, Hashimoto thyroiditis, and Graves’ disease), neurological (multiple sclerosis and myasthenia gravis), and dermatological (pemphigus vulgaris) ones, have been linked with various autoimmune cytopenias (anemia, thrombocytopenia, neutropenia, pure red cell aplasia) and also with lymphoproliferative diseases and solid cancer. They share a common underlying etiology and are caused by the impaired self-tolerance of the immune system [14,23,24,25,26]. AIHA may occur synchronously with lymphoma, may precede or follow its diagnosis for several years, and may even be related to antilymphoma therapy [24]. In addition, AIHA that is resistant to therapy is emerging in recipients who have solid organs or are recipients of hematopoietic stem cell (HCT) transplants from an unrelated HLA donor [27,28].

The relationship between AIHA and congenital conditions such as common variable immunodeficiency/hyper IgM syndrome/autoimmune lymphoproliferative syndrome/Kabuki syndrome suggest that genetic background is an important factor regarding AIHA onset. Recurrent somatic mutations of *KMT2D* and mono-allelic *CARD11* were demonstrated in patients with CAD. The loss of KMT2D function is related to the stimulation of the auto-reactive *IGHV4-34*-encoded immunoglobulin receptor, disturbed class switch recombination, and enhanced B-cell proliferation and survival. Mono-allelic *CARD11* mutations result in B-cell proliferation and auto-antibody production. Similarly, oncogenic bi-allelic *CARD11* mutations that result in constitutive nuclear factor (NF)-κB activation were demonstrated in a subset of patients with diffuse large B-cell lymphoma [16].

Conversely, the origins of autoAbs in drug-induced AIHA (DIHA) are well established. More than 160 drugs are suspected of inducing AIHA, the most commonly reported being antibiotics (penicillins, cephalosporins, and cotrimoxazole), antimycotics (fluconazole and amphotericin B), diclofenac, ibuprofen and other non-steroidal anti-inflammatory drugs, immunosuppressive (azathioprine) and antineoplastic drugs (both conventional ones like fludarabine, chlorambucil, and bendamustine, and novel options, such as immune checkpoint inhibitors), cardiovascular drugs (methyldopa, furosemide, and enalapril), and many others, including corticosteroids [2,29,30]. It is worth mentioning that some vaccines, including the mRNA-COVID 19 vaccine, have also been implicated as causes of the new onset or relapse of AIHA and Evans syndrome [31]. Although rare, DIHA encompasses about 10% of all AIHA cases [32]. There are two main subtypes of DIHA. In drug-dependent DIHA, Abs are directed to drugs adsorbed onto the RBC membrane, or to immune complexes of drug and cell membrane compounds (neoantigen). In drug-independent DIHA, IgG warm autoAbs can be detected in the absence of drugs [1]. Several drugs can modify the RBC membrane so that Ig (IgM or IgG), complement, albumin, and other plasma proteins adsorb nonimmunologically (non-immunologic protein adsorption(NIPA)), leading to a positive DAT result accompanied by slow and slight hemolysis [33].

### 3.2. Immune Clearance of Autoantibodies by Spleen Macrophages

Although an adaptive immune response to foreign antigens is initiated in the white pulp, immune effector function takes place and expands in spleen red pulp where neutrophils, monocytes, dendritic cells (DCs), gamma delta (γδ) T cells, and macrophages reside [17]. The most abundant Ig isotype in human serum and the predominant anti-RBC autoAb in wAIHA is IgG. IgG is composed of four subtypes whose constant regions differ in terms of the hinges and CH2 domains that are involved in binding to IgG-Fc receptors (FcγR) and C1q. The binding of IgG to FcγR on effector cells (macrophages, CD8+ T cells, and natural killer (NK) cells) triggers phagocytosis and antibody-dependent cell-mediated cytotoxicity (ADCC). In physiological conditions, IgG1, which is the most abundant IgG subclass, and IgG3 interact efficiently with most FcγR, while IgG2 and IgG4 show reduced affinity to a number of FcγR [34]. As a consequence, IgG1 and IgG3 shorten the RBC half-life more efficiently via ADCC than IgG2 and IgG4. In the case of high density (i.e., when two IgG molecules are in close proximity on the RBC surface), IgG1 and IgG3 can also fix C1q and activate the classical complement route, but usually not beyond C3b. The CD3b-opsonized RBCs undergo extravascular destruction caused by liver macrophages that carry receptors for C3b fragments. The downstream activation of complement cascade in wAIHA is very rare due to the monomeric IgG structure and the low Rh antigen density on RBCs. The capacity to perform terminal complement activation, the cleavage of C5, and the sequential formation of MAC (C5b-9) on the RBC surface is a feature of pentameric IgM and results in RBC agglutination and their direct and dramatic intravascular osmotic lysis, with a reported mortality rate of about 20% if the thermal activity of IgM is close to physiological temperatures [1,2,5,23,35]. The calculated speed of RBC destruction via intravascular hemolysis is 200 mL of RBCs per hour, whereas the extent of extravascular hemolysis is tenfold lesser (0.25 mL RBCs/kg/h, i.e., a patient with an average weight of 70 kg will experience a loss of 420 mL RBCs per day) [23].

Extravascular FcγR-mediated hemolysis takes place in the spleen and lymphoid organs. Spleen macrophages regulate RBC turnover in physiological and pathological circumstances. During a normal lifespan, RBCs encounter detrimental changes in plasma cell membrane and become less deformable. Senescent RBCs are too rigid to pass through the inter-endothelial slits of the spleen red pulp, and those trapped RBCs are phagocytized by macrophages located in the cords of the red pulp. Every day, approximately 1% of aged and irreversibly damaged RBCs are removed from circulation [36]. Besides the changes in deformability, over time, RBCs accumulate so-called “eat me” signals on their membrane that facilitate their clearance by macrophages. The exposure of phosphatidylserine on the outer leaflet is one of these “eat me” signals and is part of the programmed cell death process. Spleen macrophages possess receptors that recognize exposed phosphatidylserine on various cells and phagocyte them. As RBCs are non-nucleated, the term eryptocysis is used instead of apoptosis [37].

The most abundant integral membrane protein in RBCs is Band 3 (SLC4A1), with more than a million copies per cell. Band 3 is associated with a number of other membrane proteins including the Rh complex, glycophorins, and CD47. At the inner RBC membrane, Band 3 is attached to the cytoskeleton through interaction with ankyrin, while its carboxyl terminus is associated with carbonic anhydrase. Consequently, Band 3 has numerous fundamental functions in maintaining RBC integrity—from controlling RBC shape and deformability, through regulating CO_2_ transport, to mediating phagocytosis in the spleen. Namely, Band 3 exposes neoantigens that are recognized by naturally occurring Abs (Nabs) and cleared by macrophages. Conversely, CD47 represents one of the “do not eat me” signals, and binding to its receptor, signal-regulatory protein alpha (SIRPα), on macrophages suppresses phagocytosis by inhibiting the inside-out activation of integrin signaling [35]. In wAIHA, as well as in immune thrombocytopenia patients, CD47 is expressed at normal levels [38]. Spleen macrophages are not just responsible for clearing aged and damaged RBCs but also for repairing RBCs, as seen in the removal of inclusion bodies from circulating RBCs via spleen-facilitated vesiculation [39].

Splenic macrophages possess three types of FCγR receptors for the IgG heavy chain that activates phagocytosis: FcγRI (CD64), FcγRIIA (CD32a), and FcγRIII (CD16). Phagocytosed RBCs are targeted to phagolysosomes. FcγRI has the highest affinity for IgG molecules, and its activation induces signaling pathways. This includes phosphatidylinositol 3-kinase (PI3K) and mitogen-activated protein kinase (MAP), which are responsible for efficient erythrophagocytosis and a minimal release of harmful free hemoglobin into circulation. It is of clinical interest that spleen macrophages also express an inhibitory FcγRIIB and that its activation may explain the inefficiency of intravenous Ig (IVIG) in wAIHA as their therapeutic activity is mediated through binding to FcγRIIB [35].

Besides phagocytosis, spleen macrophages can only remove the IgG-coated portion of RBC membranes, resulting in a change in shape and RBCs becoming deformable. The formed rigid microspherocytes are retained in spleen red pulp sinusoids and cleared, as previously explained. Spleen CD8+ T cells and NK cells that also express FcγR are responsible for the ADCC destruction of opsonized RBCs [1,2,5,23,35].

**Figure 2:**
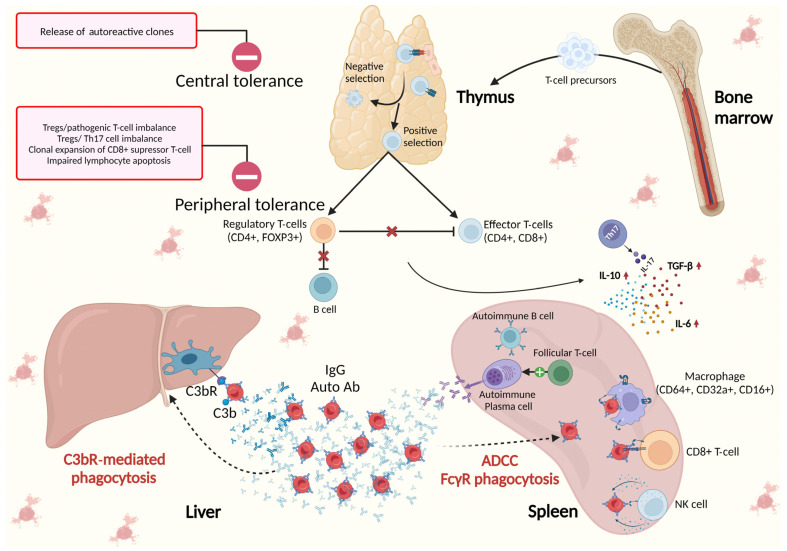
The spleen in the immunological background of AIHA. Central tolerance takes place in primary lymphoid organs (bone marrow for B-cells and thymus for T-cells) and is responsible for identifying immature self-reactive lymphocytes, i.e., T- or B-cell clones that possess receptors, to identify self-antigens with high affinity. These autoreactive clones, upon binding with self-antigens, undergo deletion or apoptosis (negative selection) or may, in the case of B-cells, change their specificity or, in the case of T cells, develop regulatory tolerance (Treg). Peripheral tolerance takes place in the germinal centers (GCs) of lymphoid follicles in secondary lymphoid organs (the spleen, lymph nodes, and mucosal lymphoid tissues) where foreign antigens are normally encountered. The importance of peripheral tolerance in preventing autoimmunity is in maintaining unresponsiveness to self-antigens that are expressed in peripheral tissues and not in primary lymphoid organs, and in preserving tolerance to self-antigens that are expressed in adult life after the production of mature lymphocytes. Peripheral tolerance is driven by specific CD4+FOXP3+ Tregs, and the imbalance between Tregs and pathogenic effector/memory CD+ T cells, as well as Tregs and Th17, together with increased clonal expansion of CD8+ suppressor T cells and impaired lymphocyte apoptosis, lead to the loss of control over T-cell activation and autoimmunity [14,23,40]. Th17 cells play crucial roles in the immune response against microorganisms and in autoimmunity and facilitate the development and progression of various cancers. They are responsible for the secretion of proinflammatory cytokine interleukin-17 (IL-17), which promotes and characterizes humoral autoimmune response, together with observed increased levels of IL-6, IL-10, and transforming growth factor (TGF)-β and reduced tumor necrosis factor (TNF)-α [15,26,40]. In contrast to normal plasma cells (PCs), autoimmune plasma cells accumulate in the spleen, establishing positive feedback with follicular T-helper cells (Tfh). Opsonized erythrocytes undergo elimination in the spleen through FcγR-mediated phagocytosis and antibody-dependent cellular cytotoxicity (ADCC), as well as in the liver via C3bR phagocytosis.

The pivotal role of the spleen in the immunological background of AIHA is illustrated in Figure 2.

### 3.3. Autoimmune Reaction against Bone Marrow and Extramedullary Hematopoiesis in the Spleen

The presence of reticulocytopenia instead of reticulocytosis is not uncommon as a sign of insufficient erythropoiesis and poor prognosis. Indeed, this is observed in 20–40% of AIHA cases [41]. It has been suggested that in the pathogenesis of AIHA, as in ITP, an autoimmune reaction against bone marrow precursor plays an important role [42]. Recently reported data from a multicenter international study on the efficacy and safety of erythropoietin (EPO) treatment in AIHA revealed that the majority of the 51 included patients had inadequate reticulocytosis and reduced endogenous EPO levels considering the degree of anemia displayed. The overall response upon receiving EPO therapy (i.e., Hb increase greater than 20 g/L) was more than 70% and was observed both in primary and secondary wAIHA and CAD/CAS, as well in treatment-naïve patients and in relapsed/refractory patients previously treated with several treatment strategies. Several possible underlying mechanisms for reduced EPO levels in AIHA have been proposed—the inability of bone marrow to promptly respond to abrupt and massive RBC destruction, the negative feedback of hypoxia to the EPO kidney production, or a state of bone marrow corresponding to that seen in sepsis [15].

It has been estimated that adult humans make more than 2.5 × 10^6^ RBCs per second. The production of RBCs is in tight balance with the turnover of senescent RBCs by red pulp macrophages in the spleen [37,43]. Paulson et al. proposed a hypothesis that, as opposed to inhibiting steady-state erythropoiesis, inflammation induces stress erythropoiesis [44]. In anemia of critical illness (ACI), steady-state erythropoiesis is impaired due to HSC exhaustion and myelosuppression. The stress erythropoiesis seen in ACI is currently being intensively investigated. It is well documented that toll-like receptor 4 (TLR4) plays a crucial role via MYD88 (myeloid differentiation factor 88) or TRIF (Toll/interleukin-1 receptor (TIR)-domain-containing adapter-inducing interferon -β) in host response to bacterial infection, mediating the innate immune response and activating signaling pathways to promote the inflammatory cascade. These effects lead to increased cytokine secretion, such as of IL-1β, IL-6β, TNFα, and NF-κB, as well as to increased neutrophil infiltration and increased apoptosis [45,46]. TLR4 is also a key regulator of chronic inflammation [47]. However, on the other hand, TLR contributes to bone marrow failure—during sepsis, MYD88 activation leads to myelosuppression, while TRIF activation compromises HSC self-renewal. Furthermore, the downregulation of Spi1 and CebpA, two key transcription factors involved in the regulation of HSC and the myeloid transcriptional program, was observed [48]. The recently published results of Noel et al. indicate that IL-1/MyD88-dependent granulocyte colony stimulating factor (G-CSF) secretion plays a key role in impairing medullary erythropoiesis [49].

Besides the removal of aged, dead, or opsonized RBCs from circulation, spleen red pulp serves as a reservoir of RBCs, with the capacity to store 15–25% of total RBC volume that can be ejected as a physiological response to hypoxia (i.e., apnea, high altitude, exercise) [50]. Furthermore, the spleen is the main organ where stress erythropoiesis occurs. In humans, the spleen is one of the major sites of hematopoiesis during late embryonic life and at birth. In adults, HCTs in the spleen at steady state are less numerous than in bone marrow, but splenic HCTs are less quiescent and have faster reconstitution ability compared to bone marrow steady-state HCTs. It has demonstrated in mice that most splenic HSC are pre-activated, i.e., in the G1 phase that facilitates their fast cell cycle entry in emergency conditions [51]. Wang et al. analyzed splenic global gene expression patterns during stress erythropoiesis and found higher levels of expression of transcriptional factors that play the primary role in RBC differentiation (*Gata1*, *Tal1*, and *Klf1*). Conversely, genes involved in the immune response were inhibited, and NK cells decreased during stress erythropoiesis [52]. Extramedullary hematopoiesis contributes to the enlargement of the spleen in AIHA.

## 4. Novel Treatment Strategies for AIHA

Current guidelines recommend glucocorticoids and rituximab for first-line therapy [5]. Transfusions of ABO-, RhD-, and K-matched blood are only warranted for patients with life-threatening wAHA (Hb level ≤ 60 g/L), as they may further promote hemolysis. Due to the thermal characteristics of cold autoAbs, transfusions to cAIHA patients should be given using an in-line blood warmer. All active AIHA patients should receive folic acid (1–5 mg/d), and vitamin B12 if deficient, as well as ensuring thromboprophylaxis with low-molecular-weight heparin [5,14,32,53]. The exacerbation of hemolysis in cAIHA can simply be prevented if patients avoid cold temperatures and warm acral areas (nose, ears, digits) [53].

Specific first-line treatment strategies for AIHA are based on the inhibition of autoAb production. Patients with newly diagnosed symptomatic wAIHA (Hb level ≤ 100 g/L) are recommended to take oral prednisone 1 mg/kg daily. In steroid-responsive patients, i.e., those whose Hb rises above 100 g/L, the taper can begin after 2–3 weeks. If the dose can be reduced to 7.5 to 10 mg per day after 3 to 6 months without the relapse of clinically significant anemia, a further tapering should be attempted until the drug is discontinued. In the case of long-term corticosteroid treatment, patients should also receive treatment to prevent osteoporosis and fragile fractures (vitamin D, calcium, bisphosphonates) [1,2,5,14,32,54,55]. The tapering of corticosteroids is gradual and cautious as only 30–40% of patients who exhibit an initial response remain in remission after one year [56]. Corticosteroids are recommended in wAIHA due to the initial response in about 80% of patients, although they are not effective in cAIHA [5,53].

In patients with wAIHA who do not respond to corticosteroids, in those who relapse during corticosteroid tapering or after discontinuation, and in those requiring unacceptably high doses (>10–15 mg daily), the introduction of rituximab is recommended. It is important to emphasize that a lack of response to corticosteroids is suggestive of secondary wAIHA and indicates further investigation. Rituximab is a chimeric human IgG1-κ mAb directed against the extracellular domain of protein CD20, which is expressed on B-cells. Initially approved for the treatment of B-cell non-Hodgkin’s lymphoma, Rituximab has proven to be safe and effective therapy for the treatment of primary and secondary wAIHA [14,57,58,59,60], and as a result, other immune suppressants (azathioprine, cyclosporine, mycophenolate, etc.) have fallen out of use, most often in wAIHA associated with systemic lupus erythematosus. Rituximab may be given in a conventional form (375 mg/m^2^ weekly for 4 weeks) or at a low dose (100 mg weekly for 4 week) and combined with corticosteroids [5,55,61]. The use of rituximab in conventional doses introduces therapeutic options for cAIHA patients requiring treatment but is less effective against cAIHA than wAIHA. As complete responses are very rare, rituximab is used in combination with cytotoxic agents such as bendamustine or fludarabine [1,53].

Better insights into the pathobiology of AIHA have encouraged the investigation of numerous agents used to inhibit immune responses at various stages. Most of them are already approved for other indications. For example, rozanolixumab, the inhibitor of neonatal Fc receptor (FcRn), shortens IgG half-life from a normal 28 days to about 10% and is currently approved for the treatment of myasthenia gravis; fostamatinib [62], the splenic tyrosine kinase inhibitor, inhibits cellular mediators of phagocytosis and reduces the clearance of RBCs and platelets and is currently approved for the treatment of ITP; bruton tyrosine kinase inhibitors ibrutinib [63,64,65,66,67,68] and acalabrutinib are currently approved for treatment of CLL, and rizabrutinib is approved for the treatment of ITP; and sirolimus [69], the inhibitor of the mechanistic target of rapamycin kinase (mTOR), is approved for the treatment of malignant perivascular epithelioid cell tumors and is also effective in the prevention of graft rejection following organ transplant [14,55,62,69,70,71,72]. Idelalisib, which is a PI3K inhibitor, and venetoclax, a selective inhibitor of the anti-apoptotic protein B-cell lymphoma 2 (Bcl-2), are emerging as promising agents for the treatment of AIHA associated with CLL [63,73].

Drugs that target LLPCs include bortezomib, a proteasome inhibitor that is already included in recommendations for the treatment of cAIHA [53] and in refractory wAIHA either alone or combined with corticosteroids and dexamethason [74,75,76,77]. Daratumumab, a mAb targeting CD38 on LLPCs, is reported to be effective in treating wAIHA following alloHSCT and severe refractory AIHA cases [78,79,80], while a study on the safety, pharmacokinetics, and efficacy of ixatusimab, another antiCD38 mAb, was recently terminated based on strategic sponsor decisions [81]. Alemtuzumab, an mAb targeting CD52, may be useful in secondary wAIHA in the context of lymphoproliferative disorders [82,83,84,85,86,87,88,89]. The results of reports of targeted therapy in patients with primary and secondary wAIHA with sufficient clinical data are summarized in Table 2 [62,63,64,65,66,67,68,69,73,75,76,77,78,79,80,82,83,84,85,86,87,88,89,90,91,92]. Molecules that target the complement cascade, such as the C3 inhibitor pegcetacoplan and the C1q inhibitor cinryze, are reported to be effective in severe complement-mediated AIHA [93,94].

## 5. The Place of Splenectomy in the Era of Novel AIHA Treatment

The enlargement of spleen in AIHA is the consequence of Ab production, the immune destruction of RBC, and extramedullary hematopoiesis, crucial factors in IHA pathobiology. The surgical removal of the spleen was recognized and widely accepted as an effective therapeutic option in corticosteroid-resistant AIHA cases a long time ago. In a study by Allgoold and Chaplin on 44 AIHA patients published in 1967, an early response was obtained after splenectomy in 68% of corticosteroid-resistant patients, and 44% were able to maintain remission without supplementary steroid therapy after more than one year post-splenectomy. On the other side, the long-range mortality rate in this cohort was 40%, and the most common cause of death was pulmonary embolism [95]. Excellent efficacy was reported two decades later in a study on 52 splenectomized AIHA patients [96], with a reported sustained complete response rate of 64% after a mean follow-up period of 33 months, minimal morbidity, and an absence of mortality. Splenectomy was confirmed to be less efficient in secondary AIHA. A retrospective review of 30 patients with AIHA who underwent splenectomy revealed significantly inferior outcomes for AIHA patients with associated diseases (the most prevalent were B-cell malignancies and rheumatologic disorders), with an overall response rate of 56% and complete remission achieved in only three out of sixteen (19%) cases, while all patients with idiopathic AIHA demonstrated a response to splenectomy that was complete in nine out of eleven (82%) instances. Of interest is the fact that the reported median time from AIHA diagnosis to splenectomy in both groups was very short, at only 3.2 months for patients with secondary AIHA, and that seven patients within this group had associated thrombocytopenia, indicating Evans syndrome, a condition that may partly explain their worse outcome [97]. The irradiation of the spleen was successfully performed in several patients with associated lymphoproliferative diseases and high perioperative risk. As splenic irradiation in this setting can be considered experimental, a total irradiation dose is not established for performing these procedures and in published cases varies from 8 Gy to 20 Gy. It is assumed that a higher radiation dose may induce a “functional” splenectomy [98].

In the years that followed, although still prior to the introduction of novel therapy in the treatment of AIHA and underling diseases, the reported median time from AIHA diagnosis to splenectomy extended and the laparoscopic procedure prevailed over the open-surgery technique [99,100]. Due to the rarity of AIHA, patients with secondary AIHA were included in most published studies. In a case series of nine patients with symptomatic and uncontrolled AIHA diagnosed concurrently with CLL, splenectomy was performed after the failure of both CLL-targeted treatment with alkylating agents and/or purine analogues and AIHA targeted treatment with IVIG and/or corticosteroids. A median time to splenectomy of 62 months was reported, with patients’ average age at the time of splenectomy being 62 years. Seven of the nine patients (78%) achieved early complete response, and six (67%) retained remission from AIHA within a mean follow-up of 24 months after splenectomy. All patients underwent splenectomy via the laparoscopic technique and seven of the nine patients (78%) experienced no immediate postoperative complications [100]. Higher response rates were achieved in patients with primary AIHA. Long-term postoperative follow-up evaluations were obtained in three studies on the efficiency of laparascopic splenectomy performed in the context of malignant and non-malignant hematologic conditions, including primary AIHA [101,102,103]. In a retrospective study of 45 ITP and 15 AIHA patients who had undergone splenectomy from 1996 through to 2010, it was observed that the complete response rate was 93%, and that all patients survived the three-year post-splenectomy period [103]. Rosen et al. reported that uncontrolled AIHA was an indication for splenectomy in nine out of one hundred and forty-seven splenectomized patients and that, after a mean follow-up period of 20 +/− 14 months’, the response rate was 89% amongst the AIHA cohort [101]. Balagué et al. reported a response rate of 70% (seven out of ten patients) over a mean post-splenectomy period of 40 ± 18 months [102]. It is also noteworthy that, when analyzing the age at which splenectomy took place, the median age of patients with AIHA was significantly higher compared to the other indications given in these three previously mentioned studies. A comprehensive review of these studies on AIHA patients with a mean age above 55 years revealed that 94% of subjects (45 out of 48) underwent laparoscopic splenectomy with a complete response of 81% at 35.6 months, while the operative mortality was assessed at 0%, with no immediate or late thrombotic events [104]. Because of the high short-term efficacy and evidence of good long-term response, splenectomy was recommended as the most effective and best-evaluated second-line therapy for all steroid-refractory or steroid-dependent AIHA patients [105].

**Figure 3:**
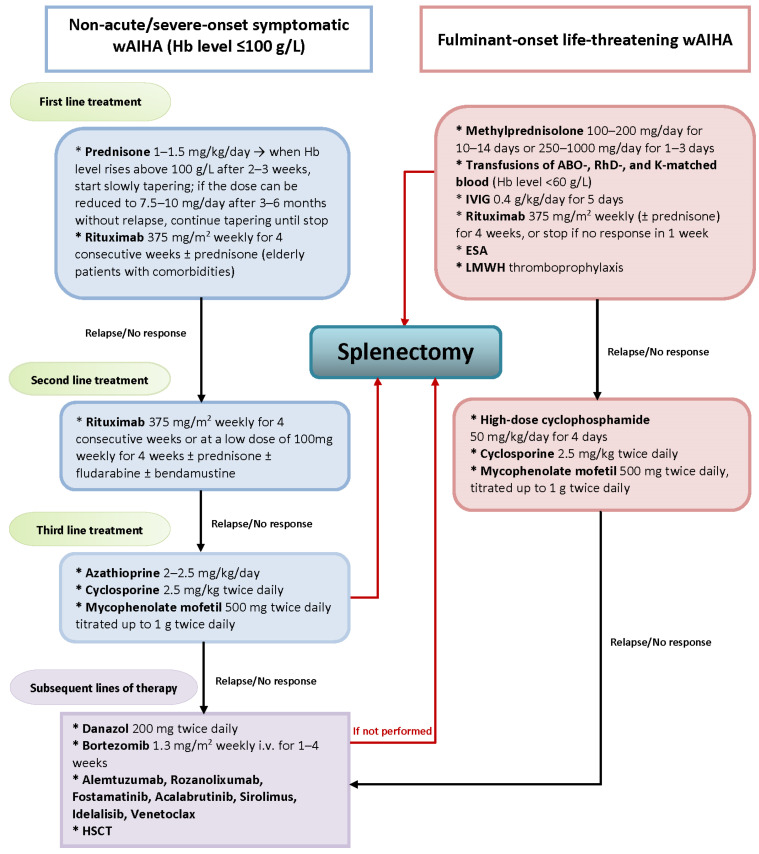
The role of splenectomy in the current treatment landscape of wAIHA. Abbreviations: wAIHA—warm autoimmune hemolytic anemia; Hb—hemoglobin; IVIG—intravenous immunoglobulin; ESA—erythropoietin stimulating agent; HSCT—hematopoietic stem cell transplantation.

The introduction of rituximab was a turning point in the treatment of AIHA and gave impetus to the use of a wide range of innovative drugs in the treatment of AIHA, relegating splenectomy to the third and subsequent therapeutic lines [5,32,54,106,107], as shown on Figure 3. In a Spanish multicenter retrospective study of 93 patients diagnosed with AIHA between 1987 and 2017, the third line of therapy, including splenectomy, was needed in only 27% of patients, and a decrease in the rate of splenectomy was observed after the successful introduction of rituximab, which was applied in 34 patients with an overall response rate of close to 100% [60].

In addition to the abovementioned study conducted by Serbian authors, which compared the safety of laparsacopic and open techniques [99], by searching the Medline database, we managed to identify three studies published in English after 2012 that related to splenectomy in AIHA adult humans, and one study related to splenectomy in adults with Evans syndrome. Maskal et al. reported a short-term complete response of 61% and a complete response of 43% after a median follow up period of 33 months in splenectomized patients with primary and secondary AIHA [108]. Barron et al. analyzed the efficacy of splenectomy in autoimmune hematological disorders in the context of systemic lupus erythematosus and found that complete remission (defined as Hb ≥90 g/L) was achieved in eleven of the sixteen patients who had secondary AIHA or Evans syndrome [109]. Of interest is a study by Ogbue et al. that reviewed a cohort of 339 adult patients with immune cytopenias who had undergone a splenectomy, including 68 patients with AIHA. Aside from the high response rate (overall response was 74%, of which 86% were complete responses) and very low rate of postoperative complications with no mortality, the finding that up to 20% of the cases had a postoperative diagnosis is of importance, as this was discordant with the original indication for splenectomy, including benign conditions, such as Felty’s syndrome, and malignant diseases, predominantly mature B-, T-, and NK-cell lymphoma [110]. This emphasizes the role of splenectomy in securing a diagnosis and a subsequent optimal treatment level. In a study by Sulpizio et al. on seven patients who had undergone splenectomy to treat Evans syndrome (two of them having been associated with hematological malignancy), an early response was achieved in 85.7% of patients, and no major surgical complications were reported, albeit with a 42.8% relapse rate and a one-year response rate of 42.8% [111]. In all four of the abovementioned retrospective studies, AIHA patients who underwent splenectomy before the introduction of rituximab were also included. We could not find any published data addressing splenectomies performed only in the rituximab era.

According to the current guidelines, splenectomy or partial splenic embolization are still recommended in the emergency management of transfusion-dependent life-threatening wAIHA that is unresponsive to prednisolone. This should be combined with other supportive therapies (erythropoietin stimulating agents (ESA) and/or plasma exchange and/or IVIG) and active immunosuppression [5,55].

The loss of crucial functions of splenic macrophages in eliminating encapsulated bacteria and prolonged antecedent or concomitant immunosuppression renders splenectomized AIHA patients highly susceptible to bacterial infections, with a reported rate of 6–7% [112]. Among the 4756 AIHA patients identified using the California Discharge Dataset 1991–2014, the cumulative incidence of sepsis was 4.3% in those who had never undergone a splenectomy, compared to rate of 6.7% who had undergone a splenectomy. For the latter group, the rate of sepsis only increased in the late postoperative period [113]. In addition to postoperative antibiotic prophylaxis, vaccination with polysaccharide and conjugate vaccines against Streptococcus pneumoniae, Haemophilus influenzae, and Neisseria meningitidis should be performed at least 14 days before a scheduled splenectomy, or after the 14th postoperative day after an urgent splenectomy [114,115]. The use of live or live, attenuated vaccines in patients receiving >10 mg/day of prednisone or a cumulative dose of >700 mg over three months should be avoided, and the Food and Drug Agency recommended deferring procedures until at least one month after steroid discontinuation [116]. This is a need that cannot always be met in the case of refractory AIHA patients.

Thromboprophylaxis is also indicated in splenectomized AIHA patients in order to minimize the risk of venous thrombosis. It has long been recognized that AIHA has a prothrombotic state, per se [117], and thromboprophylaxis is advisable and recommended for all hospitalized patients admitted for wAIHA, as well as for outpatients with marked hemolysis [5,32,54]. The risk of venous thrombosis is further increased in splenectomized AIHA patients, leading to long-term morbidity and disability [113,118,119].

Splenectomy is not recommended to treat complement-mediated intravascular hemolysis, nor is it recommended for complement-mediated extravascular hemolysis where CD3b-opsonized RBCs are destroyed via liver macrophages that carry receptors for C3b fragments [51]. This is despite the fact that several case reports demonstrating the successful management of refractory CAD with splenectomy have been published, especially in recent years [120].

## 6. Conclusions and Future Direction

The spleen is the location of origin of autoAb and the location of antibody-mediated RBC phagocytosis in AIHA, as well as the location of extramedullary hematopoiesis. Splenectomy was the therapeutic option in corticosteroid-resistant AIHA patients for decades. In the era of modern therapy, splenectomy is scarcely performed, as it is either employed as a third or subsequent line of treatment or is recommended as a rescue management technique for transfusion-dependent, life-threatening wAIHA. The reported rate of complications following splenectomy has gradually decreased over time due to less invasive laparascopic techniques, vaccination against encapsulated bacteria, and thromboprophylaxis. Today, splenectomy is not associated with increased mortality. When taking into account this and the real-world evidence of the limited wide availability of rituximab and other costly targeted therapies inspired by clinical trials in most and not only developing countries, laparascopic splenectomy holds its place as a safe and good therapeutic option in the selected pools of AIHA patients.

## Figures and Tables

**Table 1:** Serological and thermal characteristics of autoantibodies in AIHA.

AIHA Subtype		Autoantibodies				Hemolysis
		Antibody Specificity	Thermal Amplitude	Specificity	DAT	
wAIHA		IgG (+ possible C fixation)	37 °C (0–40) 98.6 °F (32–104)	Rh system glycophorins A–D	IgG or IgG + C3d	Extravascular (mainly spleen)
cAIHA	CAD	IgM, rare IgG (common C fixation)	4 °C (4–34) 39.2 °F (39.2–93.2)	I/i system, rarely Pr or IH antigen	C3d CA titer ≥64	Extravascular (liver) Intravascular
	PCH	IgG, rare IgM (common C fixation)	react at 4 °C (39.2 °F) hemolyze at 37 °C (98.6 °F)	P antigen	C3d (Donath–Landsteiner test)	Intravascular
Mixed AIHA		warm IgG and cold IgM	4 °C (39.2 °F) and 37 °C (98.6 °F)		IgG + high-titer cold IgM	Intra- and extravascular

Abbreviations: wAIHA—warm autoimmune hemolytic anemia; cAIHA—cold autoimmune hemolytic anemia; CAD—cold agglutinin disease; PCH—paroxysmal cold hemoglobinuria; IgG—immunoglobulin G; IgM—immunoglobulin M; DAT—direct antiglobulin test; C—complement; CA—cold agglutinin.

**Table 2:** Real-world evidence on the effectiveness of novel treatment strategies in patients with primary and secondary wAIHA.

Drug/Mechanism of Action	Dose Schedule	Number of Patients	Response	Time to Response	Duration of Responses	Reference
Primary wAIHA						
Fostamatinib/ syk inhibitor	100–150 p.o. mg bid	19	7/19	2–4 weeks	24 weeks	Kuter et al. 2022 [62]
Parsaclisib/ PI3Kδ inhibitor	1 mg p.o. qd 2.5 mg p.o. qd	16	75% (PR 25%)	2 weeks	12 weeks	Barcellini et al. 2022 [90]
Bortezomib/Proteasome inhibitor	1.3 mg/s.c. qw for 4 weeks + Rituximab	1	1/1	NA	9 months	Ames et al. 2010 [75]
	1.3 mg/s.c. biw for 2 weeks + Rituximab	4	4/4	1–3 weeks	3–5 months	Chen et al. 2020 [76]
	2.7 mg every 72 h total of four doses	1	1/1	1 weeks	7 months	Chineke et al. 2019 [77]
Daratumumab/ antiDC38 mAb	16 mg/kg qw for 6 weeks	1	1/1	NA	5 months	Rieger et al. 2021 [79]
	16 mg/kg qw for 4 weeks	1	1/1	12 weeks	2 months	Jain et al. 2020 [78]
	16 mg/kg qw for 2 months	1	1/1	4 weeks	NA	McGlothlin et al. 2022 [80]
Sirolimus/ mTOR inhibitor	1–3 mg/d 6–18 months	14	12/14 (4 PR)	12–24 weeks	16 months	Li et al. 2020 [69]
Secondary wAIHA						
Alemtuzumab/ antiCD52 mAb wAIHA+CLL	10 mg qd for 1.5 weeks	2	2/2 (1 PR)	8 weeks	16 months	Willis et al. 2001 [83]
	30 mg d5, then 30 mg tiw for 3 weeks	1	1/1	3 weeks	10 months	Rondon et al. 2003 [84]
	3 mg d1, 10 mg d3, and 30 mg tiw for 8 weeks	1	1/1	NA	16 months	Cheung et al. 2006 [85]
	10 mg s.c. d1, then 30 mg tiw for 8 weeks	1	1/1	8 weeks	15 months	Lundin et al. 2007 [86]
	30 mg s.c. tiw 3–12 w for 8 weeks	5	5/5	5 weeks (4–7)	15 months	Karlsson et al. 2007 [87]
	30 mg tiw: 8 weeks i.v.—episode 1 11 weeks s.c.—episode 2	1	1/1	eeks—episode 1 NA—episode 2	17 months (episode 1) 3 months (episode 2)	Royer et al. 2007 [88]
	3 mg d1-d3, then 10 mg tiw for 10–13 weeks	3	3/3	5–8 weeks	9–26 months	Leurenti et al. 2007 [89]
	after dose escalation, 30 mg tiw 8–16 weeks	3	3/3	NA	7–68 months	Mc Alister et al. 2019 [82]
Idelalisib/ PI3Kδ inhibitor	150 mg p.o.bid + Rituximab	12	11/12 (8 PR)	NA	median PFS not reached after 2 years	Quinquenel et al. 2019 [63]
Ibrutinib/ BTK inhibitor wAIHA+ CLL	420 mg p.o. qd +/− Rituximab	16	15/16 (8 PR)	NA	median PFS 19 months	Quinquenel et al. 2019 [63]
	420 mg p.o. +/− Rituximab	8	6/8	NA	17.6 months	Vitale et al. 2016 [64]
	420 mg p.o. qd + Prednisone 12.5 mg p.o. qd for 4 weeks	1	1/1	5 weeks	12 months	Cavazzini et al. 2016 [65]
	420 mg p.o. + oral corticosteroids 6–17 weeks	5	5/5	6–10 weeks	15–150 weeks	Garcia-Horton A et al. 2016 [66]
	420 mg + Prednisone ≤ 20 mg qd *	21	NA	NA	17.5 months	Montilo et al. 2017 [67]
Ibrutinib/ BTK inhibitor wAIHA+ MCL	560 mg p.o. qd	1	1/1	7 weeks	6 months	Galinier et al. 2017 [68]
Venetoclax/ Bcl-2 inhibitor wAIHA+CLL	after standard ramp up, 400 mg p.o. qd	1	1/1	3 months	10 months	Lacerda et al. 2017 [91]
	after standard ramp up, 400 mg p.o. qd	1	1/1	4 weeks	6 months	Gordon et al. 2019 [92]
	after standard ramp up, 400 mg p.o. qd + Rituximab	2	2/2	3 months	13 mo and 29 months	Galindo-Navarro et al. 2023 [73]

Abbreviations: wAIHA—warm autoimmune hemolytic anemia; FcRn—neonatal Fc receptor; mAb—monoclonal antibody; syk—splenic tyrosine kinase; BTK—bruton tyrosine kinase; PI3Kδ -phosphatidylinositol 3-kinase δ inhibitor; mTOR—mammalian target of rapamycin; Bcl-2—B-cell lymphoma 2; CLL—chronic lymphocytic leukemia; MCL—mantle cell lymphoma; PR—partial response; i.v.—intravenous: s.c.—subcutaneous; p.o.—orally (per os); qd—once daily; bid—twice daily; qw—once weekly; biw—twice weekly; tiw—three times weekly; NA—not applicable * patients with uncontrolled AIHA requiring >20 mg prednisone daily were excluded from the RESONATE study.

## Data Availability

Not applicable.
